# On the natural diversity of phenylacylated-flavonoid and their in planta function under conditions of stress

**DOI:** 10.1007/s11101-017-9531-3

**Published:** 2017-09-08

**Authors:** Takayuki Tohge, Leonardo Perez de Souza, Alisdair R. Fernie

**Affiliations:** Max-Planck Institute of Molecular Plant Physiology, Am Mühlenberg 1, 14476 Potsdam-Golm, Germany

**Keywords:** Flavonoid, Natural diversity, Phenelyacylation, Species conservation, UV-B

## Abstract

Plants contain light signaling systems and undergo metabolic perturbation and reprogramming under light stress in order to adapt to environmental changes. Flavonoids are one of the largest classes of natural phytochemical compounds having several biological functions conferring stress defense to plants and health benefits in animal diets. A recent study of phenylacylated-flavonoids (also called hydroxycinnamoylated-flavonoids) of natural accessions of Arabidopsis suggested that phenylacylation of flavonoids relates to selection under different natural light conditions. Phenylacylated-flavonoids which are decorated with hydroxycinnamoyl units, namely cinnamoyl, 4-coumaroyl, caffeoyl, feruloyl and sinapoyl moieties, are widely distributed in the plant kingdom. Currently, more than 400 phenylacylated flavonoids have been reported. Phenylacylation renders enhanced phytochemical functions such as ultraviolet-absorbance and antioxidant activity, although, the physiological role of phenylacylation of flavonoids in plants is largely unknown. In this review, we provide an overview of the occurrence and natural diversity of phenylacylated-flavonoids as well as postulating their biological functions both in planta and with respect to biological activity following their consumption.

## Introduction

Flavonoids are by far the major sub-class of phenylpropanoids, which are themselves the third largest class of plant secondary metabolites (specialized metabolites). Flavonoids comprise in excess of 8000 metabolites bearing a common diphenylpropane (C_6_–C_3_–C_6_) backbone (Tohge et al. 2013a, b). Several classes of flavonoids have been categorized on the basis of their core skeleton: C_6_–C_3_–C_6_ (flavonoids), (C_6_–C_3_–C_6_)_2_ (biflavonoids) and (C_6_–C_3_–C_6_)_n_ (condensed tannins). The major flavonoids, in which two aromatic rings are linked via a three carbon chain (C_6_–C_3_–C_6_), have been reported to have manifold biological functions in the productive pollen fertility, signalling with microorganisms, auxin transport regulation and pigmentation, as well as in the protection of plants from various environment stresses, for example high light, UV-B irradiation and pathogens/pests (Taylor and Grotewold 2005; Tohge et al. 2013a, b; Nakabayashi et al. 2014). Additionally they exhibit a range of diverse functions including roles in developmental processes in plants (Taylor and Grotewold 2005; Silva-Navas et al. 2016; Tohge and Fernie 2016). On the other hand, flavonoids have several health beneficial effects including anti-retroviral, anti-hypertensive, anti-inflammatory, anti-aging and insulin-sensitizing activities, the reduction of the risk of a range of chronic diseases including cardiovascular disease, cancer and osteoporosis as well as inhibition of low-density lipoprotein (LDL) oxidation (Butelli et al. 2008; Crozier et al. 2009; Zhang et al. 2014; Tohge and Fernie 2016).

**Fig. 1:**
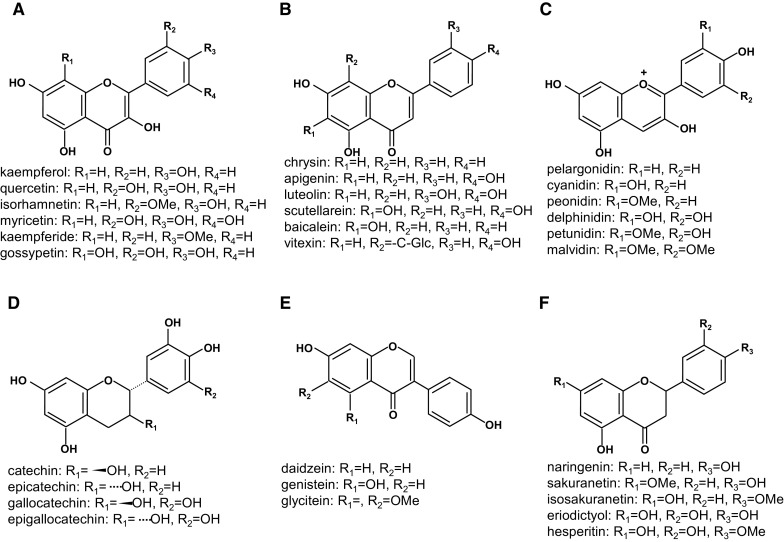
Major flavonoid aglycones in land plants. **a** Flavonols, **b** flavones, **c** anthocyanidins, **d** flavanols, **e** isoflavones and **f** flavanones

Some sub-classes of phenylpropanoids, such as syringyl-lignin and hydroxycinnamate biosynthesis are consequently widespread in plant lineages even in Angiosperms. On the other hand, some flavonoid biosynthetic genes are missing in several plant species such as algae and moss (Tohge et al. 2013a, b). Since orthologous genes of the early steps in flavonoid pathway are found even in liverworts and mosses (Winkel-Shirley 2001), the evolution of flavonoid biosynthesis appears to be as yet incompletely understood. However, the appearance of subclasses of flavonoids (Fig. 1), suggests that branches in the pathway have evolved in specific plant lineages over the course of evolution. The core flavonoid biosynthetic pathway is well documented. The main chemical backbone of flavonoids (flavonoid aglycones, Fig. 1) is synthesized from phenylalanine via a set of core biosynthetic genes including CHS, chalcone isomerase (CHI), flavanone 3-hydroxylase (F3H), dihydroflavonol 4-reductase (DFR), anthocyanidin synthase (ANS) that are relatively well-conserved among land plants. Specific steps make specialized flavonoids such as isoflavone synthase (IFS) and isoflavone reductase (IFR) which make isoflavones, flavonol synthase (FLS) required for flavonol biosynthesis and flavonoid 3′,5′-hydroxylase (F3′5′H) for production of 3′,5′-hydroxylated flavonoids. Six major types of flavonoid aglycones, flavonols (Fig. 1a e.g. kaempferol and quercetin), flavones (Fig. 1b e.g. apigenin and luteolin), anthocyanidins (Fig. 1c e.g. cyanidin, delphinidin and petunidin), flavanols (Fig. 1d e.g. catechin and epicatechin), isoflavones (Fig. 1e e.g. daidzein) and flavanones (Fig. 1f e.g. naringenin and sakuranetin) are the major sub-classes of the flavonoid family. In addition, additional steps (Zhang et al. 2014) are involved in further decoration such as glycosylation and acylation of flavonoids and these exhibit a large natural diversity and are commonly species-specific (Tohge et al. 2013a, b). Several gene families have been characterized functionally as being involved in flavonoid decoration. Flavonoid glycosyltransferase genes were found in the UDP-sugar-dependent glycosyltransferase 1 (UGT1) (Yonekura-Sakakibara and Hanada 2011) and glycoside hydrolase family 1-type (beta glucosidase, BGLU) (Matsuba et al. 2010; Ishihara et al. 2016) gene families. Similarly, flavone synthases (FNS-I and FNS-II) have been found from both P450 and 2-oxoglutarate/Fe(II)-dependent dioxygenase (2-ODD) gene families (Tohge et al. 2013a, b). Furthermore, flavonoid acyltransferases have been characterized from both the BEAT/AHCT/HCBT/DAT (BAHD) (D’Auria 2006; Tohge et al. 2015; Petersen 2016) and the serine carboxypeptidase-like (SCPL) (Tohge et al. 2016) gene families. Interestingly, the same or highly similar products can be formed by enzymes from different gene families using differently-activated substrates. Such evolutionary convergence of flavonoid decoration enzymes has been subject to natural selective pressures which generate mechanisms for tolerance to environmental stress such as that imposed by natural UV-B (280–315 nm) irradiation (Jenkins 2009; Tilbrook et al. 2013) and photo-oxidative damage induced by photo-inhibition (Steyn et al. 2002;). Such effects of irradiance in UV-B wavelength range on plants has been widely studied, since UV-B causes primary effects of non-specific damage to DNA, RNA, proteins and lipid membranes as well as secondary effects such as inhibition of photosynthesis and growth, induction of cell death, and deformities in flower and pollen morphologices. Plants have a UVR/COP/HY5 cascade light signalling system to protect them from harmful UV-B damage, which includes metabolic perturbation and reprogramming under light stress. Part of this metabolic reprograming induces production of phytoprotectants such as phenylpropanoids in land plants. Enhancing our understanding of gene conservation and the corresponding specificity of flavonoids in nature is important for future studies in which interesting natural variants are characterized at the molecular genetic, biochemical and physiological levels to lay the foundations for the design of stress resistant crop plants.

Natural chemical diversity of functional phytochemicals often corresponds to the evolution of adaptation to specific environments, and therefore the understanding of natural diversity using a broad range of accessions offers a very powerful tool to investigate phytochemical functions in nature. Whilst studies of natural variation and diversity using accessions have been carried out in many plant species, including Arabidopsis, with regard to the accumulation of specific primary metabolites and glucosinolates (Schauer and Fernie 2006; Rowe et al. 2008; Carreno-Quintero et al. 2013; Wen et al. 2014; Schilmiller et al. 2015), few examples have focused on flavonoid biosynthesis (Routaboul et al. 2012; Ishihara et al. 2016; Tohge et al. 2016).

In a recent study, we isolated phenylacylated-flavonols amongst natural accessions of Arabidopsis and characterized the gene encoding flavonol phenylacyltransferase 2 (AtFPT2, At2g22960) responsible for the production of accession specific phenylacylated-flavonols. Additionally our UV-B stress experiments using transgenic plants expressing FPT2 and the gain-of-function NIL suggested that phenylacylation of flavonoids relates to the response to UV-B stress (Tohge et al. 2016). Phenylacylated-flavonoids are decorated with several hydroxycinnamoyl units (cinnamoyl, 4-coumaroyl, caffeoyl, feruloyl and sinapoyl). Furthermore, computational estimation of the most stable stereochemical structure of 6″-*O*-phenylacylflavonol resulted in a bridge piled structure between the aromatic rings A and sinapoyl ring like intramolecular copigmentation of phenylacylated anthocyanins (Bakowska-Barczak 2005; Tohge et al. 2016). Given that phenylacylation confers enhanced UV-absorbance, understanding the natural diversity in phenylacylated-flavonoids might provide insights into adaption to UV-B irradiance.

Phenylacylated-flavonoids are widely distributed in plant species including vegetables, trees and ornamental flowers. Genes encoding flavonoid-phenylacyltransferases were found in some of these species. These genes are known to have emerged via convergent evolution i.e. they have evolved independently in species of different lineages, but have highly similar functions. In this review, we detail recent advances in our understanding of (1) UV-B responses linked to the production of flavonoids, (2) studies of natural variance in the response to UV-B, (3) function of specific decorations of flavonoids under UV-B stress, and (4) broader natural diversity of phenylacylated-flavonoids in the plant kingdom.

### Consequences of climate change on UV-B irradiance

Given a booming global population, a stable food supply under changing environmental conditions is becoming a priority, globally. The risk of UV-B damage is fundamental, although only 0.5% of UV-B (280–315 nm) irradiance reaches the earth’s surface (Blumthaler 1993). As we described above, UV-B causes several primary and secondary effects on organisms. When DNA absorbs UV-B energy, cyclobutane pyrimidine dimers (CPDs) form, leading to mutations (Garinis et al. 2005). In addition, proteins and lipids are damaged by UV-B irradiation itself (Kramer et al. 1991; Hideg et al. 2013). Oxidative stress, caused by an imbalance between reactive oxygen species (ROS) production and antioxidant scavenging capacity have been proposed as key factors in UV-B stress (Jenkins 2009). An overabundance of ROS production additionally damages cell components, resulting in apoptosis. The thylakoid membrane has been proposed as the source of free radicals generated from hydrogen peroxide (H_2_O_2_) cleavage in response to UV-B light (Hideg and Vass 1996), although the precise mechanism of ROS generation following exposure to UV-B irradiation remains unclear (Yannarelli et al. 2006; Jenkins 2009). ROS mediated signaling acts by adjusting gene expression, proteolysis and thioredoxin dynamics.

Given the profound effects of light that plants are subjected to, considerable selective pressure to generate tolerance mechanisms involved in both sensing and responding to natural UV-B irradiation (Sablowski et al. 1994; Jenkins 2009; Kusano et al. 2011; Tilbrook et al. 2013). Since the fractionation of UV-B irradiation after passing through the atmosphere is largely dependent on atmospheric conditions (for example cloud cover, snow, aerosol etc.), the final intensity of UV-B reaching the earth´s surface is highly unstable and variant day to day, although the total area of the ozone hole has decreased slightly since 2006 as a result of efforts to restrict the use of chlorofluorocarbons (Solomon et al. 2016). Such instability of UV-B irradiation could therefore impair crop yields greatly, since the detrimental effects described above combine to constrain food security considerably by impacting stabilities of food supplies, crop morphology and plant and seed yields (Mohammed and Tarpley 2010; Wargent and Jordan 2013).

### The UV-B signalling response is mediated by the UVR8/COP/HY5 pathway

**Fig. 2:**
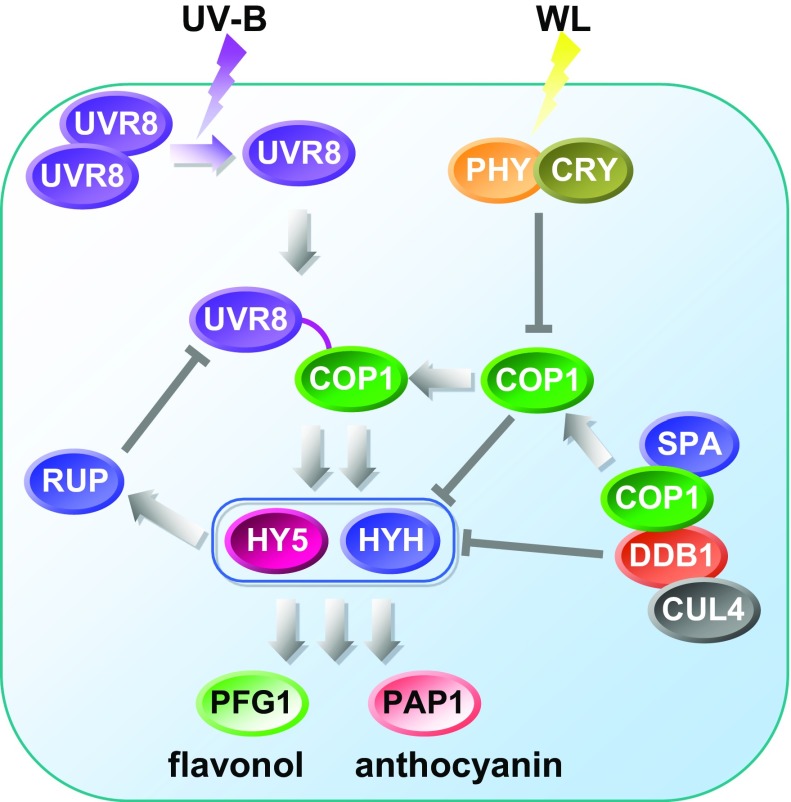
Model of UVR8/COP1 mediated UV-B signaling cascade. Current model of UVR8/COP1 mediated signaling cascade. *PHY* PHYTOCHROME A, *CRY* CRYPTOCHROME, *UVR8* UV REPAIR DEFECTIVE 8, *COP1* CONSTITUTIVE PHOTOMORPHOGENIC 1, *DDB* UV-damaged DNA binding protein, *HY5* ELONGATED HYPOCOTYL 5, *HYH* HY5-HOMOLOG, *CUL4* CULLIN 4, *RUP* REPRESSOR OF UV-B PHOTOMORPHOGENESIS

Our understanding of the signaling following UV-B irradiation in plants has been greatly enhanced by work on the UV-B photoreceptor of Arabidopsis (*Arabidopsis thaliana*) (AtUVR8) and the downstream signaling cascades it triggers (Rizzini et al. 2011). Current models of the signal transduction and transcriptional regulation network of the UV-B response in higher plants are summarised in Fig. 2a. UV-B photoreceptors have been studied in several plant species including desert poplar (Mao et al. 2015), grapeberry (Liu et al. 2015) and apple (Zhao et al. 2016). Interestingly a similar UVR8-mediated UV-B perception and acclimation response has been characterized functionally in Chlamydomonas (Tilbrook et al. 2016) suggesting that this response evolved early in the green plant lineage.

The genes involved in the UV-B signaling cascade have largely been characterized by forward genetic screening for UV-B irradiation-sensitive mutants. The principal genes identified as mediators of UV-B photomorphogenic responses are CONSTITUTIVE PHOTOMORPHOGENIC 1 (COP1, encoding a multi-functional E3-ubiquitin ligase) (Oravecz et al. 2006), ELONGATED HYPOCOTYL 5 (HY5, encoding a bZIP transcription factor) (Ulm et al. 2004), HY5-HOMOLOG (HYH) (Brown and Jenkins 2008) encoding another bZIP transcription factor and UV-B photoreceptor UVR8 (Rizzini et al. 2011). Interestingly, UV-B regulates the expression of many genes involved in diverse processes including metabolism, morphogenesis, photosynthesis, UV-protection and pest and pathogen defense (Kilian et al. 2007; Findlay and Jenkins 2016). Within this signalling cascade, the UV-B photoreceptor, UVR8, acts to up-regulate both HY5 and HYH, and down-regulate expression of COP1 which can balance photo-morphogenesis and high light responsive pathways. By contrast, the transcription factors, HY5 and HYH, control several MYB regulators such PRODUCTION OF ANTHOCYANIN PIGMENT 1 (PAP1/AtMYB75) and PRODUCTION OF FLAVONOL GLYCOSIDES (PFG1/MYB12) for anthocyanin and flavonol pathways respectively (Fig. 2a) (Tohge et al. 2005; Stracke et al. 2011). In addition, the UV-B responsive AtMYB4 was characterized as a negative regulator of phenylpropanoid pathway by its function in downregulating expression of the genes encoding cinnamate-4-hydroxylase (C4H) and 4-CoA ligase (4CL) which are key enzymatic steps in general phenylpropanoid metabolism in Arabidopsis. Expression of AtMYB4 represses the production of sinapoyl-malate which offers protection against UV irradiation. AtMYB4 belongs to quite an ancient family of genes encoding R2R3MYB transcriptional repressors. It may be that the earliest UV protecting agents were hydroxycinnamic acid-esters, such as chlorogenic acids. In specific cases, flavonoids may have taken over some or all of the roles of UV-protection but not in species belonging to the family Solanaceae, like tobacco which relies heavily on chlorogenic acids for UV-B protection. These regulators which are, in turn, under the regulation of HY5 suggested that the complex structure of light signalling cascade can influence metabolism in order to adapt to severe environments of higher UV-B intensity.

### The metabolic hierarchy of the UV-B response

Despite the direct connections within the UV-B signaling cascade between UV-B irradiation and metabolic reprogramming, signaling has been reported to adopt a hierarchic multi-phasic response structure in several plant species (Casati et al. 2011; Kusano et al. 2011; Kim et al. 2012; Kaling et al. 2015). Indeed the reprogramming of metabolic pathways appears to contain many different factors including ROS effects, inhibition of photosynthesis, the imbalance of energy and central metabolism and large-scale changes in gene expression but also the production of phytochemical protectants. During the early stages of exposure to UV-B irradiation, plant cells are “primed” for higher primary metabolite levels via a mechanism involving metabolic reprogramming for efficient conservation of carbon to the aromatic amino acid (AAA) precursors for the production of phenylpropanoids such as hydroxycinnamates and flavonoids. Metabolite profiling over a time course following UV-irradiation allow us to suggest that ascorbate plays an important role in the short-term response to UV-B irradiation. Additionally, results suggested that both flavonoid and hydroxycinnamates play a more important role in the adaptive response of plants to UV-B exposure (Kusano et al. 2011). Observation of general reductions in sugar levels (Casati et al. 2011; Kusano et al. 2011; Kim et al. 2012) and the reduced expression of genes associated with photosynthesis, photorespiration and the Calvin–Benson cycle (Kilian et al. 2007) allowed us to suggest a consequent down-regulation of genes associated with cell wall synthesis, starch synthesis and lipid metabolism (Koch 1996; Kusano et al. 2011). On the other hand, the transcriptomic and metabolomic changes in the TCA cycle, nucleotide metabolism and amino acid biosynthesis suggested these changes are only partially in accordance with changes in the steady-state levels of intermediates which are closely associated with these processes.

### Natural variance in the response to UV-B

Although many studies have reported the UV-B protective function of various phenylpropanoids focusing on the biological processes such as flower development, pollination and seed production (Rozema et al. 2009), few studies succeeded to establish any relationship between UV-B stress and different accessions. In a recent study of growth related traits in 345 *Arabidopsis thaliana* accessions in response to UV radiation stress, a mutation in phenylalanine ammonia lyase (PAL1) which encodes the first enzyme of phenylpropanoid biosynthesis was found to be associated with genetic polymorphism between UV-B sensitive and resistant accessions (Piofczyk et al. 2015). Interestingly the Arabidopsis pna-10 accession was characterized as a natural deletion mutant of the SNG1 (At2g22990) gene, which encodes an enzyme in the biosynthesis of sinapoyl-malate which is one of hydroxycinnamate derivatives (Li et al. 2010).

**Fig. 3:**
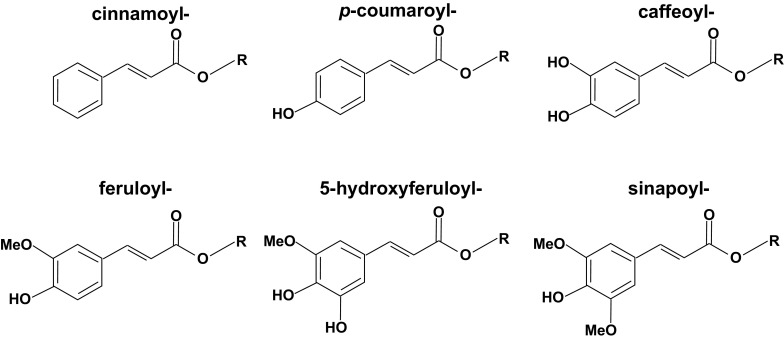
Phenylacyl moieties found in flavonoids

Recently we performed profiling of natural variation of floral secondary metabolite composition and abundance in 64 *Arabidopsis* accessions (Tohge et al. 2016). For this purpose a previously described liquid-chromatography/mass-spectrometry (LC/MS) protocol (Tohge and Fernie 2010), was used to profile 68 peaks which included 24 flavonoids and 18 unknown peaks which were putatively annotated as flavonol derivatives, by MS/MS analysis. Following purification of these compounds a battery of analytical chemical techniques including 1- and 2-D nuclear magnetic resonance (NMR) spectroscopy was employed, and one of the major peaks was identified as a novel phenylacylated-flavonol glycoside (flavonol-3-*O*-(2″-*O*-rhamnosyl-6″-*O*-sinapoyl)glucoside-7-*O*-rhamnoside) and given the trivial name saiginol A (Tohge et al. 2016). The other 17 peaks, subsequently named saiginiols B-R, were annotated as novel phenylacylated-flavonol glycosides (flavonol-3-*O*-(-6″-*O*-phenylacyl)glycoside-7-*O*-rhamnosides) according to the presence or absence of three possible aglycones (Fig. 1, kaempferol, quercetin and isorhamnetin) and as displaying one of three types of phenylacylation (Fig. 3, sinapoyl, caffeoyl and *p*-coumaroyl moieties) based on MS/MS fragmentation and elution time profiles. A total of 29 putative flavonol-phenylacylglycosides (annotated to date are 2″- or 4″-*O*-phenylacylated-flavonols) have been documented in Brassica vegetables (Cartea et al. 2011).

Following mQTL (metabolic quantitative trait locus) analysis using Near Isogenic Lines (NIL) harboring chromosome segmental substitution of Col-0 (saiginol non-producer) in C24 (saiginol producer) or the reciprocal substitutions of C24 in Col-0, and transcriptome analysis focusing solely on the mQTL region, only two genes were identified that displayed massively altered expression. These genes were subsequently renamed as putative *flavonol phenylacyltransferases* (*AtFPT*). The second candidate gene, FPT2 (At2g22960), was functionally characterized by metabolite profiling of *35S*-driven overexpressing transgenic plants (Tohge et al. 2016). Sequence analysis revealed short or large gene deletions (2–600 bp) in non-producer accessions. Interestingly, the accessions deriving from high irradiance habitats (from latitudes between 16 and 43° North or altitudes above 1000 m) produced saiginols. Additionally phenylacylation renders flavonols much more efficient in UV-B absorption than their molecular precursors. Importantly, the levels of only the saiginols and not the overall levels of phenylpropanoids were altered in these accession demonstrating the speicificity of this modification. These results suggested that FPT2 and by implication the saiginols confer additional protection against UV-B. In further UV-B treatment experiments using transgenic plants expressing *FPT2*, and the gain-of-function NIL, saiginol producing lines showed higher silique production and seed production under UV-B irradiation (Tohge et al. 2016). This result suggested that phenylacylation renders flavonoids much more efficient in UV-B absorption and tolerance under UV-B irradiation.

### Other plant phenylacyltransferase genes

Genes encoding flavonoid phenylacyltransferases which transfer hydroxycinnamoyl moieties to flavonoid glycosides were first identified within the BAHD gene family. The BAHD gene super family includes a large number of acyltransferases using CoA activated acids. Phenylacyl-CoA: anthocyanin-3-*O*-phenylacyltransferase activities were reported, at an early stage, in some plant species such as *Silene dioica* (Kamsteeg et al. 1980) and *Matthiola incana* (Teusch et al. 1987). Subsequently, anthocyanin-5-*O*-Glc-6‴-*O*-phenylacyltransferase in *Gentiana triflora* (Fujiwara et al. 1998) and *Perilla frutescens* (Yonekura-Sakakibara et al. 2000) and anthocyanin-3-*O*-rhamnosylglucoside *p*-coumaroyltransferase in *Iris hollandica* (Yoshihara et al. 2006) were identified. In Arabidopsis, two genes encoding anthocyanin-3-*O*-glucoside *p*-coumaroyltransferases were characterized (Luo et al. 2007). Recently flavonoid-3-*O*-rutinoside-4‴-phenylacyltransferase (SlFdAT1, Solyc12g088170) was identified using a combined metabolomics and transcriptomics integrative approach using *Del*/*Ros1* expressing transgenic purple tomato (Tohge et al. 2015).

In contrast, the AtFPT2 gene corresponding to saiginol production found in Arabidopsis accessions encodes a serine carboxypeptidase-like (SCPL) gene (Tohge et al. 2016). Amongst SPCL genes only the phenylacyltransferase AtFPT2 and AtSAT (anthocyanin-3-*O*-(2″-*O*-xylosyl)glucoside *p*-coumaroyltransferase (Fraser et al. 2007) have been characterized to date. Both AtFPT2 and AtSAT genes are found within genomic regions recently evolved via tandem duplication of SCPL genes. Flavonoid phenylacyltransferase genes are conserved only within closely related species but not between plant species having similar functions of phenylacyltransferase. Interestingly, both BAHD and SCPL genes which could be encoding phenylacyltransferase are found not only in flavonoid biosynthesis but also in biosynthesis of other UV-B protectants such as sinapoyl-derivatives in Brassica plants and chlorogenic acids in Solanaceous plants. A hydroxycinnamoylCoA: quinate hydroxycinnamoyl transferase (HQT) which is responsible for the synthesis of chlorogenates in Solanaceous plants is an anciently evolved BAHD enzyme. HQT and a hydroxycinnamoyl-CoA transferase (HCT) stand out from other BAHD acyl-transferases (Sonnante et al. 2010) as having evolved early and retained their structural differences over long periods of evolutionary time unlike the other more versatile BAHDs. Despite of this fact, Brassica phenylacyltransferase genes are assumed to have relatively recently evolved under natural selective pressure such as light selection following their speciation.

### Natural diversity of phenylacylated-flavonoids in plants

**Fig. 4:**
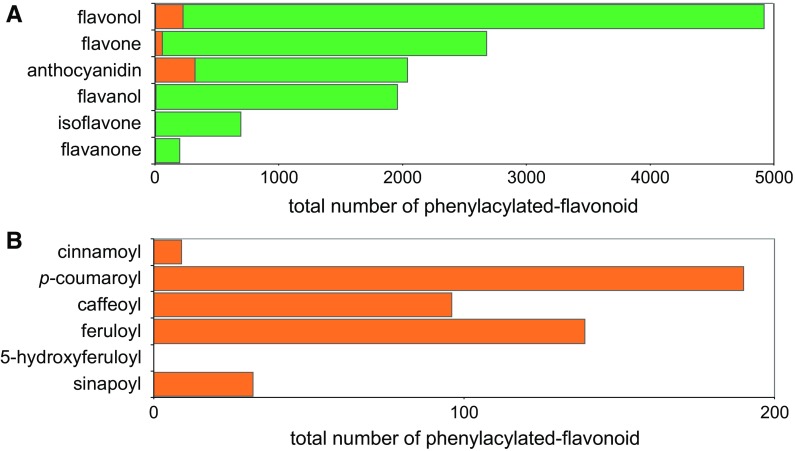
Natural diversity and ratio of flavonoid with/without phenylacyl moieties in land plants. **a** Total number of flavonoid and phenylacylated-flavonoid in each sub-class of flavonoids, **b** total number of phenylacylated-flavonoid in each decoration type. Flavonoid entries found in KNApSAcK (http://kanaya.naist.jp/knapsack_jsp/top.html) were used. *Green* and *orange* indicate non-phenylacylated- and phenylacylated-flavonoid, respectively. (Color figure online)

**Fig. 5:**
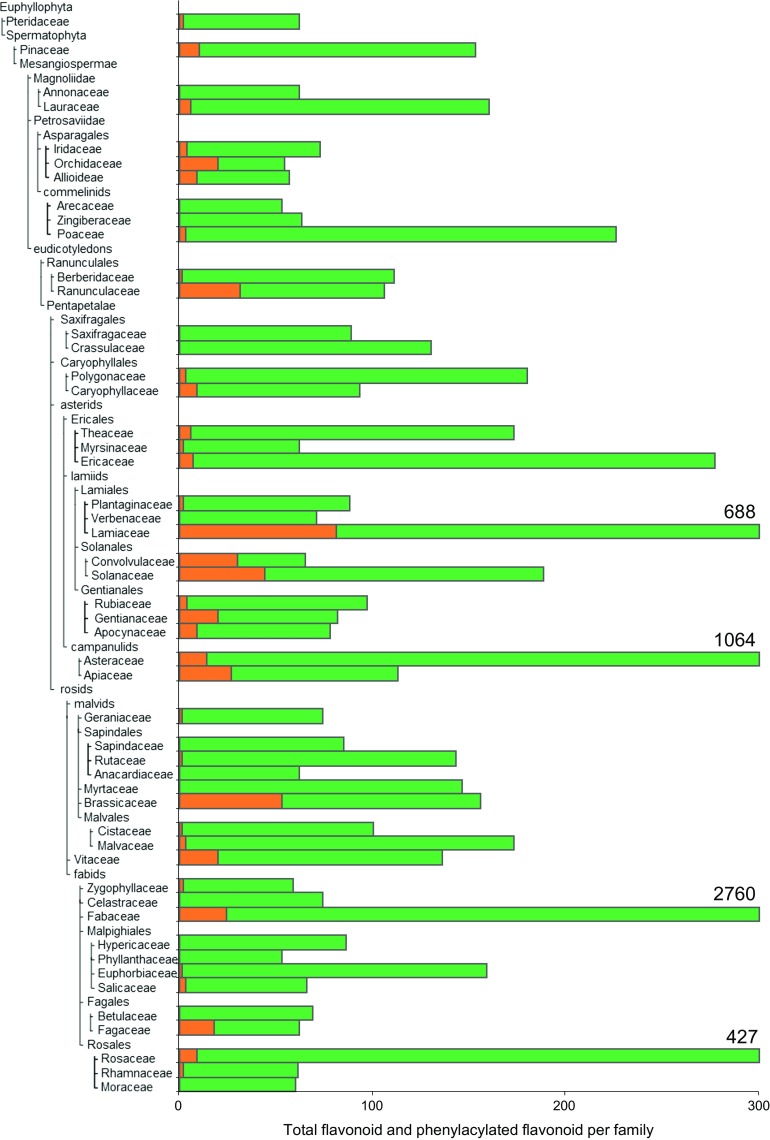
Natural diversity and ratio of total flavonoid and phenylacylated flavonoid per family in land plants. Flavonoid entries found in KNApSAcK (http://kanaya.naist.jp/knapsack_jsp/top.html) were used

Given the species specificity of phenylacyltransferase genes, the understanding of natural diversity of phenylacylated-flavonoids has become an important means by which to study the relationship between gene functional- and chemical-diversities in nature. In order to check natural diversity of phenylacylated-flavonoids, the KNApSAcK database (http://kanaya.naist.jp/KNApSAcK/) (Afendi et al. 2012) which is one of the largest databases of known literature based phytochemicals was used. Major flavonoids were searched for aglycones and phenylacyl moieties described in Figs. 1 and 3. A total of 2846 flavonoids in 293 plant species were found (total 12,469 pairs of flavonoids per species). Interestingly, flavonols (4.7% of 4914 compounds) and anthocyanins (18.5% of 2038 compounds) exhibited a much higher proportion of phenylacylation than other classes of flavonoids, flavone (2.0% of 2673 compounds), flavanol (0.3% of 1959 compounds), isoflavone (0.1% of 690 compounds) and flavanone (0.0% of 195 compounds) (Fig. 4a). Phenylacylated-flavonoids are, therefore, mainly found in conserved classes of flavonoids such as anthocyanins and flavonols. Additionally *p*-coumaroyl, caffeoyl and feruloyl moieties are the major decoration form for phenylacylation whilst no 5-feruloyl-flavonoids [only one fond in purple lisianthusa by our literature survey (Markham et al. 2000)] were found amongst currently reported compounds (Fig. 4b). The comparisons of flavonoids and phenylacylated-flavonoids by compound class or decoration type showed clear differences between compound classes. However, in comparisons of total flavonoids and phenylacylated-flavonoids per species, no clear correlation between the taxonomic tree or speciation was observed (Fig. 5). Solanales, Gentianales, Campanulids and Brassicaceae plants exhibit a relatively high ratio of phenylacylated-flavonoids, but their closely related species did not share this trend. Our analysis of the natural diversity of phenylacylated-flavonols suggests that flavonoid-phenylacylation has likely evolved for certain flavonoid classes independently in different plant species. We contend that further understanding of natural diversity of phenylacylated-flavonoids and functional characterization of phenylacyltransferases will provide an overview of phytochemical evolution in relation to plant speciation and natural selections such as light selection.

## Conclusions

To adapt under light stress conditions, land plants have a light signaling system including induction of flavonoid production which can mitigate light stress. In a recent study of natural accessions of Arabidopsis, phenylacylation of flavonoids was found to confer enhanced phytochemical functions as UV-B protectants in plants. In our analysis of natural diversity of phenylacylated-flavonoids, this type of flavonoid decoration was found in some specific flavonoid class such as anthocyanin and flavonol. Additionally they were observed in several specific plant species. Given the fact that we assume this is due to the functional convergence of phenylacyltransferase then the identification of genes encoding flavonoid-phenylacyltransferases and studies of natural diversity of phenylacylated-flavonoids will be highly valuable in order to secure food yields. However functional characterization of phenylacylflavonoids are still not well documented, and the investigation of associations of phenylacylation of flavonoids with other stress conditions such as where there are limitations on dark fixation of photosynthesis but incident light is high, as in cold, high light conditions, will thus be an important topic for future research. Additionally since flavonoids offer a legion of other protective functions, both in planta and following dietary intake by animals, it will be important to assess the role of phenylacylated flavonoids in these contexts.

## Abbreviations

ANS: Anthocyanidin synthase
BAHD: BEAT/AHCT/HCBT/DAT
BGLU: Beta glucosidase
CHI: Chalcone isomerase
CHS: Naringenin-chalcone synthase
COP1: CONSTITUTIVE PHOTOMORPHOGENIC 1
CPD: Cyclobutane pyrimidine dimers
CRY: CRYPTOCHROME
CUL4: CULLIN 4
DDB: UV-damaged DNA binding protein
DFR: Dihydroflavonol 4-reductase
EBG: Early biosynthetic genes
F3′5′H: Flavonoid 3′,5′-hydroxylase
F3H: Flavanone 3-hydroxylase
FLS: Flavonol synthase
FPT: Flavonol phenylacyltransferase
HY5: ELONGATED HYPOCOTYL 5
HYH: HY5-HOMOLOG
IFR: Isoflavone reductase
IFS: Isoflavone synthase
LBG: Late biosynthetic genes
LDL: Low-density lipoprotein
NIL: Near isogenic lines
NMR: Nuclear magnetic resonance
PAL: Phenylalanine ammonia lyase
PHY: PHYTOCHROME A
QTL: Quantitative trait locus
RUP: REPRESSOR OF UV-B PHOTOMORPHOGENESIS
SCPL: Serine carboxypeptidase-like
UGT1: UDP-sugar-dependent glycosyltransferase 1
UV: Ultra-violet
UVR8: UV REPAIR DEFECTIVE 8
